# The Microstructure and Corrosion Resistance of Fe-B-W-Mn-Al Alloy in Liquid Zinc

**DOI:** 10.3390/ma15031092

**Published:** 2022-01-30

**Authors:** Zixiang Luo, Ke Liu, Zizhen Cui, Xuemei Ouyang, Chen Zhang, Fucheng Yin

**Affiliations:** 1 School of Material Science and Engineering, Xiangtan University, Xiangtan 411105, China; 201921001459@smail.xtu.edu.cn (Z.L.); czz2012nino@sina.com (Z.C.); fuchengyin@xtu.edu.cn (F.Y.); 2 Key Laboratory of Materials Design and Preparation Technology of Hunan Province, School of Material Science and Engineering, Xiangtan University, Xiangtan 411105, China; 3 Jianglu Machinery and Electronics Group Co., Ltd., Xiangtan 411100, China; liuke820x@126.com (K.L.); ZC719500@163.com (C.Z.)

**Keywords:** Fe-B-W alloy, manganese, aluminum, molten zinc, microanalysis, corrosion

## Abstract

The microstructure, interfacial characteristics, and corrosion resistance of Fe-W-Mn-Al-B alloys in molten zinc at 520 °C have been investigated using scanning electron microscopy (SEM), X-ray diffractometry (XRD), and electron probe micro-analysis (EPMA). The experimental results indicate that the Fe-B alloy with 11 wt.% W, 7 wt.% Mn, and 4 wt.% Al addition displays a lamellar eutectic microstructure and excellent corrosion resistance to molten zinc. The toughness of M_2_B-type borides in the hyper-eutectic Fe-4.2B-11W-7Mn-4Al alloy can be more than doubled, reaching 10.5 MPa·m^1/2^, by adding Mn and Al. The corrosion layer of the Fe-3.5B-11W-7Mn-4Al alloy immersed in molten zinc at 520 °C comprises Fe_3_AlZn_x_, δ-FeZn_10_, ζ-FeZn_13_, and η-Zn. The lamellar borides provide the mechanical protection for α-(Fe, Mn, Al), and the thermal stability of borides improves as the fracture toughness of the borides increases, which jointly contribute to the improvement of the corrosion resistance to the molten zinc.

## 1. Introduction

Hot-dip galvanizing is one of the more effective and economical methods to treat steel against atmospheric corrosion, and it has been widely used [1,2,3,4]. Nevertheless, the severe corrosion in continuous galvanizing line (CGL) processing is a challenge for the long-term submersed hardware [5,6,7,8]. The corrosion seriously decreases the service life of the equipment immersed in the molten zinc bath, which further affects the production efficiency and product quality. Hence, it is necessary to search for excellent corrosion-resistant materials under elevated temperatures from 450 to 600 °C in molten zinc.

In recent years, some studies on corrosion-resistant materials in CGL have been conducted, and many kinds of materials have been used in the immersed hardware, including ceramics, Stellite alloys, intermetallics, coatings, and other composite materials [9,10]. Among these materials, because of the unique non-wetting characteristic of the Fe_2_B phase in molten zinc and their reticular structure, the Fe-B alloys display remarkable anti-corrosion properties and have drawn much research interest [11,12,13,14,15]. Ma et al. [11] investigated the effect of B on the corrosion resistance of the Fe-B alloy, finding that the Fe-B alloy containing 3.5 wt.% B showed the optimal corrosion resistance. They also found that the thermal stability of the Fe_2_B phase decreased significantly once the temperature of molten zinc reached or went above 520 °C, promoting the spalling of the borides and corrosion failure [12]. Alloying methods are widely used to improve the toughness of borides and the corrosion resistance of alloys. The effects of Cr, Mn, W, Mo, Ti, rare earth, and other elements on the fracture toughness and hardness of Fe_2_B in Fe-B-C cast alloy have been investigated [16,17], while the effect of Cr, Ni, W, Mo, and other elements on the corrosion resistance of Fe-B alloys in molten zinc baths was reported upon [18,19,20,21]. Recently, Jian et al. [16] investigated the effect of Mn addition on the microstructure, mechanical properties, and wear behavior of an Fe-3.0B alloy, finding that proper Mn addition could improve the toughness of Fe_2_B. Liu et al. [20] reported that adding 11 wt.% W to the Fe-3.5B alloy could significantly increase its corrosion resistance to molten zinc by the improvement of boride stability and through the grain refining of eutectic borides. However, the brittle characteristics of the boride phase and the preferential corrosion of the α-Fe phase remain dominant reasons for corrosion failure, which cause difficulties in meeting (CGL) industry requirements. In addition, based on the previous studies [22,23], the Fe-Al intermetallic layers could obstruct the diffusion of Zn atoms, and thereby delay the reaction between the Fe substrate and molten zinc. Al can improve the hardness and red hardness of steel, which is beneficial for improving the wear resistance of steel at elevated temperatures. Al can form a highly stable and protective oxide scale, which can improve the corrosion resistance of steel at elevated temperatures [24]. Therefore, Mn and Al were chosen as the alloying elements in the present work. In this study, we investigate the further improvement of the corrosion resistance of an Fe-3.5B-11W alloy in molten zinc by adding proper Al and Mn. The microstructural evolution of the Fe-B alloys with proper Mn and Al addition was characterized. Special attention was paid to the synergistic effects of Mn and Al on the corrosion behaviors of the Fe-3.5B-11W alloy in the molten zinc.

## 2. Experimental Procedures

### 2.1. Specimens Preparation

The raw materials are 99.9 wt.% iron ingot, 99.9 wt.% pure W, 99.9 wt.% pure Mn, 99.9 wt.% pure Al, and an FeB alloy which contains 19.0–21.0 wt.% B, less than 0.15 wt.% C, and less than 1.0 wt.% Si according to the GB/T 5682-2015 Ferro Boron. Firstly, the Al-Mn master alloy is prepared by vacuum melting to reduce the evaporation of Al. As known by the Al-Mn phase diagram [25], a large amount of Al can be dissolved in βMn. The melting point of the Mn-Al alloys increases with the addition of Al compared with that of pure Al. Then, pure Fe, W, FeB alloy, and Al-Mn master alloy were melted together by vacuum melting. Four cast Fe-B alloys containing about 3.5 wt.% B, 11 wt.% W, 7 wt.% Mn, and 0–6 wt.% Al were prepared through orthogonal tests. The compositions of these four Fe-B alloys are given in Table 1.

### 2.2. Morphology and Phase Characterization of the Specimen

All specimens embedded in resin were polished to a mirror finish using Al_2_O_3_ and etched with 4 vol.% nital solution. The surface and cross-sections of the as-cast and corroded alloys were observed using an optical microscope and JSM-6360LV scanning electron microscope (JEOL, Tokyo, Japan) equipped with an energy dispersive spectrometer. An JXA-8230 electron probe microanalyzer (JEOL, Tokyo, Japan) and a wavelength-dispersive X-ray spectrometer (WDX) were employed to determine the phase chemical composition. The constituent phases were further identified with an X-ray diffractometer (XRD, Rigaku Ultima IV, Tokyo, Japan) with Cu Kα radiation as the X-ray source, and performed in continuous scanning mode at 40 kV and 40 mA. The corroded samples were ground carefully to remove the pure zinc layer.

### 2.3. Corrosion Test in Molten Zinc

The corrosion depth profiles were used to characterize the average corrosion rate. The original thickness of each specimen was measured with a microscale micrometer 10 times at multiple locations across the cross-section of the specimen to obtain average values. The corrosion tests were executed in a graphite crucible placed in a vertical resistance furnace with the temperature maintained at 520 ± 3 °C for 24, 48, 72, 96, and 120 h. Then, these specimens were removed from the molten zinc and cooled quickly by water quenching to preserve the microstructure at that temperature. The average corrosion depth and corrosion rate of the tested specimen were calculated with the following Equations (1) and (2) [18,19,20,21]:

H = (a − b)/2(1)

R = h/t(2)

where h is the average corrosion depth (μm), a is the original thickness (μm), b is the final thickness (μm), R is the corrosion rate (μm·h^−1^), and t is the corrosion time (h).

### 2.4. Vickers Micro-Indentation Fracture Toughness Test

The micro-hardness of the boride phase in the Fe-B alloy was measured using an MH-5L Vickers hardness tester with a load of 200 g, according to the ASTM E384 standard [26]. Each value was obtained from the average of five measured values. The fracture toughness can be calculated with Equation (3) [14,15,16,17]:

K_c_ = XP/c^3/2^ (3)

X = 0.064(E/H)^1/2^ (4)

where X is the residual indentation coefficient, which depends on the hardness-to-modulus ratio (E/H) of the boride. E and H are the Young’s modulus and micro-hardness, respectively. Additionally, the value of E is approximately 336 GPa [27] for the fracture toughness calculation. P is the applied load and c is the indentation half crack length.

## 3. Results

### 3.1. Microstructural Characteristics

Four as-cast Fe-B alloys containing approximately 3.5 wt.% of B, 11 wt.% of W, 0–7 wt.% of Mn, and 0–6 wt.% of Al, were prepared through orthogonal testing. The microstructure and corrosion resistance of the four alloys are described and discussed below.

**Figure 1:**
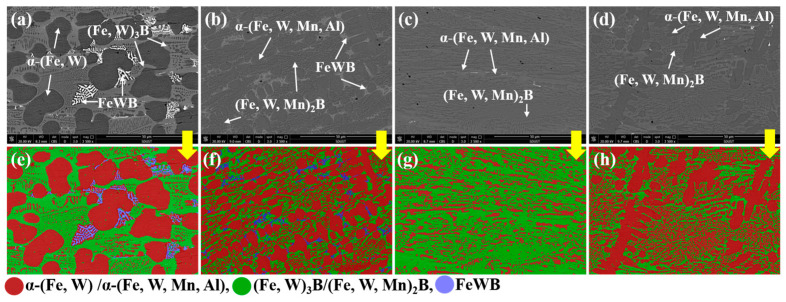
The as-cast microstructures and phase distribution of alloys: (**a**,**e**) alloy Fe-3.5B-11W; (**b**,**f**) alloy Fe-3.5B-11W-7Mn-1Al; (**c**,**g**) alloy Fe-3.5B-11W-7Mn-4Al; (**d**,**h**) alloy Fe-3.5B-11W-7Mn-6Al.

**Figure 2:**
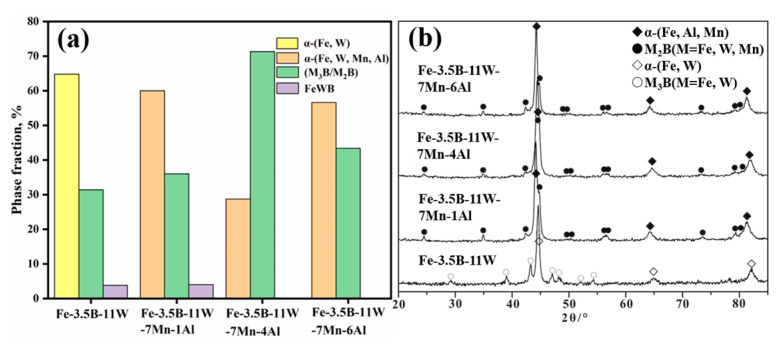
(**a**) The calculated phase fractions of the alloys; (**b**) the XRD patterns of alloys.

The microstructure of the four as-cast alloys and the constituent phases based on the WDX and XRD results is shown in Figure 1, and the XRD patterns are shown in Figure 2b. The microstructure of alloy Fe-3.5B-11W is a typical hypoeutectic morphology consisting of the primary solid solution, a gray, net-like M_3_B and white FeWB, as shown in Figure 1a. Compared with alloy Fe-3.5B-11W, the gray, net-like M_3_B-type boride changed to a net-like M_2_B-type boride, and the amount of FeWB was reduced. As the addition of Al further increases, the microstructure of the alloy Fe-3.5B-11W-7Mn-4Al changes to the almost pure finer eutectic structure without the primary solid solution. Alloy Fe-3.5B-11W-7Mn-6Al containing 6 wt.% Al has a typical hypoeutectic morphology consisting of the primary solid solution, a gray, net-like M_2_B. Firstly, the white, binary eutectic FeWB disappears (L→FeWB+α), which can eliminate the effect of destroying the integrity of the reticular borides. The size of the primary solid solution becomes small, and the primary solid solution disappears in alloy Fe-3.5B-11W-7Mn-4Al. Compared with other alloys, the eutectic borides in alloy Fe-3.5B-11W-7Mn-4Al exhibit a lamellar structure, and the microstructure is significantly finer. The software Image-Pro Plus was used to calculate the phase fraction, and the results are shown in Table 2. The microstructure treated by three different colors is shown in Figure 1e–h, in which the red phase is the α solid solution, the green one is M_3_B and M_2_B, and the blue one is FeWB. 

The calculated phase content in the alloys is shown in Figure 2a. The results indicate that the content of M_2_B increases first and then decreases, and that of α decreases first and then increases with the addition of Fe-3.5B-11W. Alloy Fe-3.5B-11W-7Mn-4Al has most M_2_B borides and less of the primary α-(Fe, Mn, Al) phase. According to the composition results of the WDX analysis (see in Table 2), the Fe-3.5B-11W atoms are mainly distributed in the α solid solution, while most Mn and W atoms are present in the borides. Compared with alloy A1, the addition of Fe-3.5B-11W and Mn can promote the formation of M_2_B-type borides and restrain the formation of M_3_B-type borides and FeWB. As a consequence, the addition of Fe-3.5B-11W and Mn can affect the solidified microstructure and promote the formation of dense borides, which benefits the improvement of corrosion resistance to the molten zinc.

### 3.2. Micro-Hardness Testing

**Figure 3:**
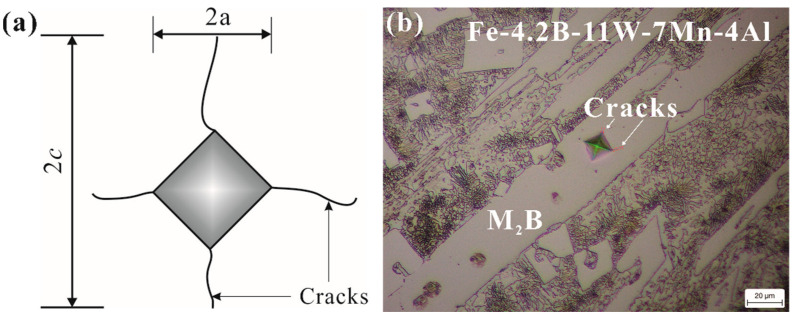
(**a**) Schematic of indentation mark and crack length; (**b**) micrographs of indentation marks and cracks on M_2_B in Fe-4.2B-11W-7Mn-4Al.

The toughness is a key factor in the stability of borides. We investigated the effects of Mn and Al addition on the fracture toughness and hardness of Fe_2_B; however, the borides in alloy Fe-3.5B-11W-7Mn-4Al were too small for fracture toughness testing via Vickers micro-indentation. Therefore, alloy Fe-4.2B-11W-7Mn-4Al, with a hypereutectic composition, was designed to obtain the block Fe_2_B phase. A schematic of the indentation mark and crack length, as well as an optical micrograph of the micro-cracks in the M_2_B, are shown in Figure 3. The average micro-hardness of Fe_2_B in alloy Fe-4.2B-11W-7Mn-4Al is 1565 HV_0.1_, somewhat higher than that of Fe_2_B (~1552 HV_0.1_) and (Fe, Mn)_2_B (1560 HV_0.1_), and lower than that of (Fe, W)_2_B (>1570 HV_0.1_). The average value of the fracture toughness is approximately 10.5 MPa·m^1/2^, compared to the fracture toughness values of Fe_2_B at 3.8 MPa·m^1/2^, (Fe, W)_2_B at 6.9 MPa·m^1/2^, and (Fe, Mn)_2_B at 4.7 MPa·m^1/2^ [15,16]. It can be concluded that the addition of Mn and W to Fe_2_B benefits the improvement of the fracture toughness. The radius of the Mn atom (1.32 Å) and W atom (1.41 Å) is larger than the Fe atom (1.27 Å), which may introduce more impact to the surrounding B atoms and bond energy in the [002] B-B. The primary weak B-B bond is expected to be strengthened naturally by the addition of the Mn and W elements, which is consistent with the results calculated by Zhou et al. [28]. 

### 3.3. Corrosion Kinetics

**Figure 4:**
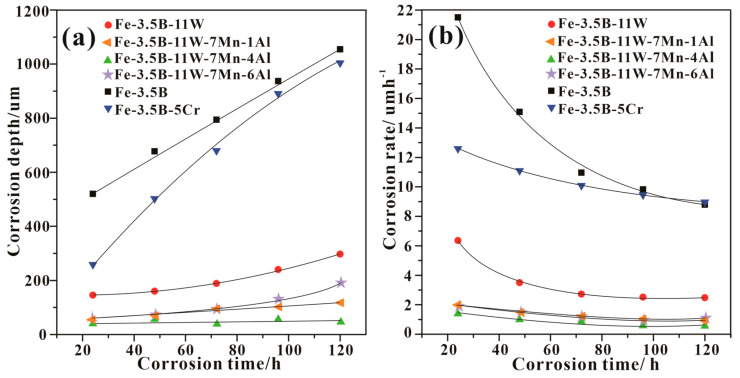
Corrosion depth and corrosion rate as a function of corrosion time at 520 °C: (**a**) corrosion depth vs. corrosion time; (**b**) corrosion rate—corrosion time.

The corrosion behaviors of these four as-cast alloys in the molten zinc were investigated with a corrosion test. Alloy Fe-3.5B-11W was selected as the standard against which to compare corrosion resistance. Figure 4 shows the corrosion depths and corrosion rates as a function of corrosion time based on Equations (1) and (2). From Figure 4a, it can be seen that the corrosion depth of all the Fe-B alloys increases with increasing immersion time. The Fe-3.5B-11W-7Mn-4Al alloy shows the best corrosion resistance among the various alloys, including alloys Fe-3.5B-11W, Fe-3.5B-11W-7Mn-1Al, Fe-3.5B-11W-7Mn-4Al, Fe-3.5B-11W-7Mn-6Al, Fe-3.5B, and Fe-3.5B-5Cr. From Figure 4b, it can be seen that all investigated samples show declining corrosion rates with increased immersion time. Meanwhile, alloy Fe-3.5B-11W-7Mn-4Al shows a comparatively low corrosion rate (<2 μm·h^−1^) among these four kinds of Fe-B alloys. 

### 3.4. Corrosion Layer Characterization

**Figure 5:**
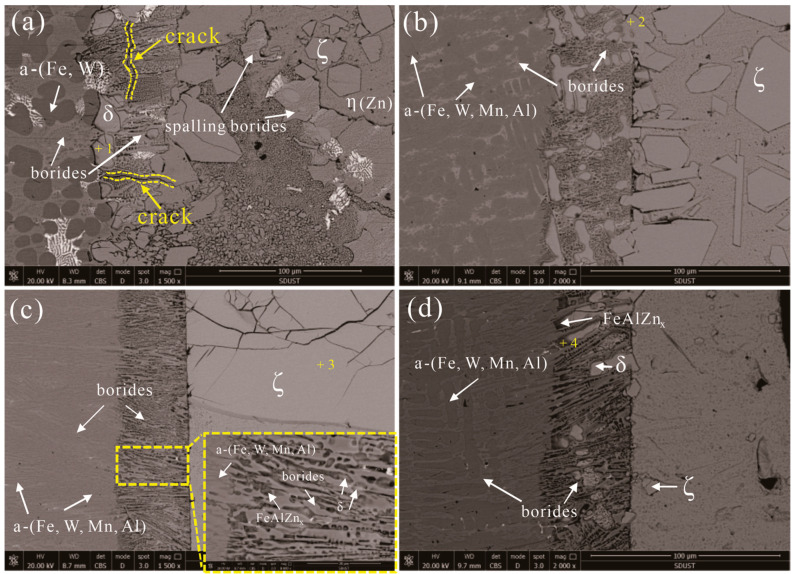
The morphology of the corrosion cross-section after corrosion testing at 520 °C for 48 h: (**a**) alloy Fe-3.5B-11W; (**b**) alloy Fe-3.5B-11W-7Mn-1Al; (**c**) alloy Fe-3.5B-11W-7Mn-4Al; (**d**) alloy Fe-3.5B-11W-7Mn-6Al.

The cross-sectional morphologies of the corrosion interface of the samples corroded by the molten zinc at 520 °C for 48 h are illustrated in Figure 5. The cross-sections of these corroded samples can be divided into three different regions: The un-corroded matrix, the corrosion layer, and the Fe-Zn compound layer. Generally, the corrosion interfaces are not uniform, and the reticular structure is destroyed by molten zinc, except in alloy Fe-3.5B-11W-7Mn-4Al. The corrosion layer mainly comprises the borides and Fe-Zn compounds. The WDX analysis results of the Fe-Zn and Fe-Al-Zn intermetallic com pounds, measured at each point 10 times, are given in Table 3.

**Figure 6:**
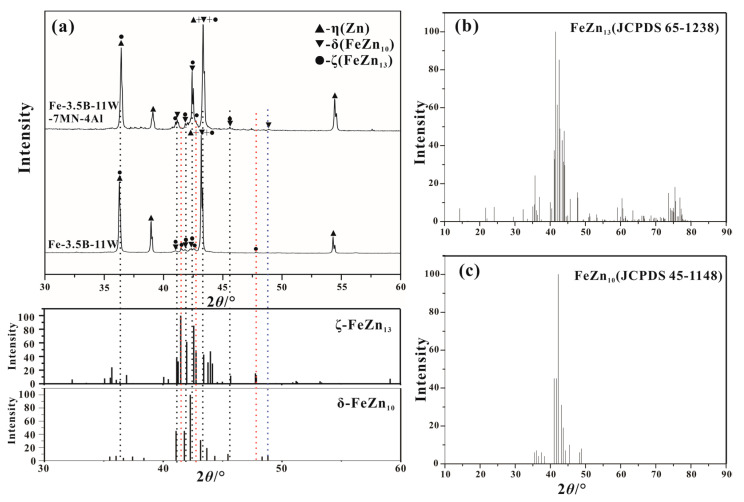
The XRD patterns of the corrosion products in alloys Fe-3.5B-11W and Fe-3.5B-11W-7Mn-4Al.

The Fe_3_AlZn_x_ alloy was found to be close to the matrix, as shown in Figure 5d, which was not confirmed by the XRD because the content is too small. The Fe-Al-Zn intermetallic compounds were observed at the corrosion interface of the Fe-B alloy to be immersed in liquid 0.25 wt.% Al-Zn [12]. The main phases in the corrosion layer are identified to be δ-FeZn_10_, ζ-FeZn_13_, and η-(Zn), which are identified by the XRD shown in Figure 6. The δ-FeZn_10_ phase is distributed on the right side of the corroded layer, near to the Fe-Zn compound layer, while the ζ-FeZn_13_ phase presents dispersed blocks in the molten zinc. There are many cracks in the δ-FeZn_10_ phase, which provide the channel for the diffusion of zinc atoms. Moreover, little spalling of the borides is found in the three alloys, which demonstrates that both alloys have good corrosion resistance to the molten zinc, but alloy Fe-3.5B-11W-7Mn-4Al is superior to alloy Fe-3.5B-11W. 

**Figure 7:**
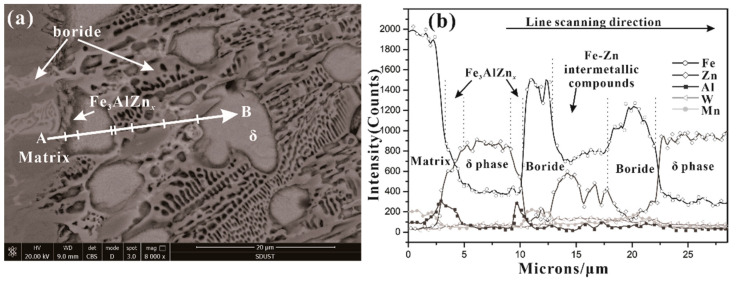
Line scanning results of element distribution near the matric and corrosion layer in alloy Fe-3.5B-11W-7Mn-1Al after corrosion testing at 520 ℃ for 48 h: (**a**) line scan SEM image; (**b**) line scan element distribution results.

The element distribution of the corrosion layer near the matrix in alloy Fe-3.5B-11W-7Mn-1Al is shown in Figure 7. It can be seen that the Fe-3.5B-11W element is indeed enriched at the interface between the matrix and the corrosion diffusion layer. Mn atoms mainly enter into the borides because of the similar atomic radius (R_Mn_ = 0.132 nm, R_Fe_ = 0.127 nm) and electro-negativities ($X_{P}^{\mathrm{Mn}}=1.55$, $X_{P}^{\mathrm{Fe}}=1.83$) of Mn as a replacement for Fe in borides. It is related to the smaller W diffusion coefficient and smaller solubility in the molten zinc pool. It can be seen from the distribution of Zn in the boride phase that the solubility of Zn is very low, which is the reason why the boride resists molten zinc corrosion. Due to the greater diffusion coefficient of zinc (D_Zn_ > D_Fe_) in the zinc bath, zinc atoms easily diffuse to the corrosion interface along the grain boundaries of Fe-Zn compounds. Subsequently, a few zinc atoms enter the (Fe, W, Mn)_2_B lattice. The fracture toughness of (Fe, W, Mn)_2_B is higher than (Fe, W)_2_B, which delays the formation of the crack. Moreover, Al diffuses in preference to Fe atoms, and dissolves at the interface between α-Fe and the borides, leading to the formation of Fe_3_AlZn_x_. 

## 4. Corrosion Mechanism

**Figure 8:**
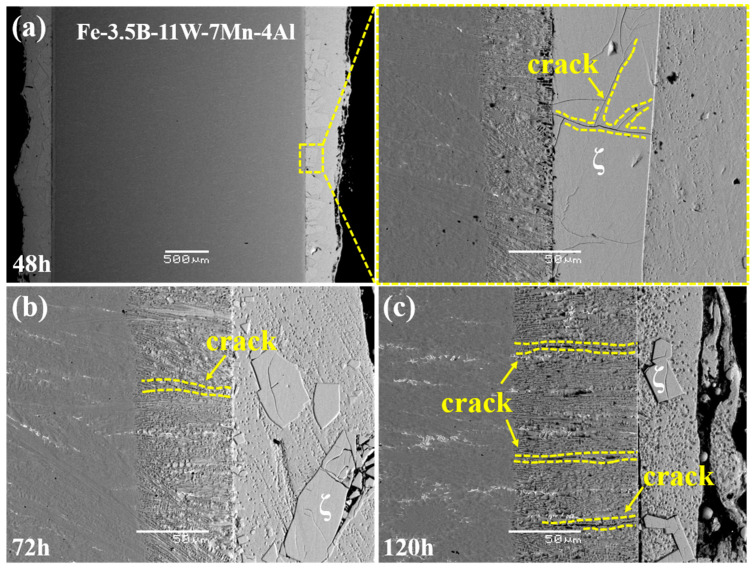
The microstructure of the corrosion layer of the alloy Fe-3.5B-11W-7Mn-4Al immersed in the zinc for different times: (**a**) 48 h; (**b**) 72 h; (**c**) 120 h.

**Figure 9:**
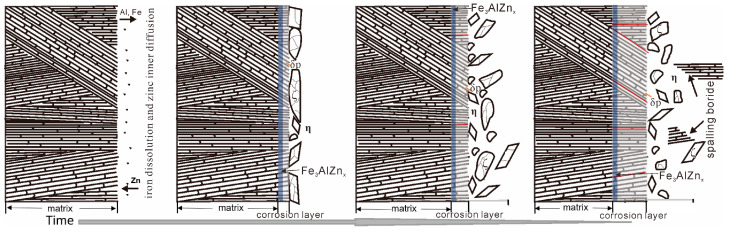
The schematic diagram of the whole corrosion behavior of alloy Fe-3.5B-11W-7Mn-4Al in liquid zinc.

The microstructure of the corrosion layer of the Fe-3.5B-11W-7Mn-4Al alloy immersed in the zinc for 48 h, 72 h, and 120 h is shown in Figure 8. The alloy Fe-3.5B-11W is enriched at the interface, forming Fe_3_AlZn_x_, and promoting the formation of the bulky ζ-FeZn_13_, as shown in Figure 8a. No microcracks were found in the alloy corroded for 48 h, and the chunky ζ-FeZn_13_ with a lot of cracks forms at the front end of the interface, and the interface close to the η-Zn is uniform, as shown in Figure 8b. With the increase in corrosion time, the size of the ζ-FeZn_13_ become small and the number of the cracks at the corrosion layer increases, as shown in Figure 8c,d. Meanwhile, most cracks grow perpendicular to the corrosion interface, which can delay the fracture of boride. In addition, few cracks were found on the δ-FeZn_10_ alloy following the addition of the Al element compared with that of Fe-B alloys reported [11,12]. With the increase in the number of cracks on the boride, the alloy eventually fails with the continuous spalling of the boride. Since the spalling boride moves with the flowing zinc, it is difficult to observe the spalling boride at the interface of the samples. In order to visually describe the entire corrosion process, the whole corrosion behavior of the Fe-W-Mn-Al-B as-cast alloy in liquid zinc can be represented schematically in Figure 9. 

Based on the observations above, the main reasons for this good corrosion resistance should be the lamellar eutectic structure, the increase in fracture toughness and volume of the borides, and the preferred growth direction of the Fe_2_B perpendicular to the corrosion interface. Alloy Fe-3.5B-11W-7Mn-4Al comprises the eutectic structure with no primary solid solution phase, which corrodes first and transforms to the brittle δ-FeZn_10_. The lamellar structure of the borides provides the mechanical protection for α-(Fe, W, Mn, Al). The alloying elements dissolve into molten zinc to promote the growth of the bulky ζ-FeZn_13_ and delay the diffusion of Zn atoms to the matrix [27]. With the increase in time, the size of ζ-FeZn_13_ becomes small and the effect slows down. The high Al dissolution in solid solution can reduce the activity of Zn and then retard the reaction of Fe and Zn [12,29]. Because the fracture toughness of borides is increased with the Mn and Al addition, the thermal stability of the borides improves, the cracks in the borides decrease, and boride spalling is caused by the transformation stress of Fe-Zn intermetallics and the attack from molten zinc is efficiently reduced [30,31,32]. In addition, the orientation of M_2_B has a great effect on the corrosion behavior of Fe-B alloys in molten zinc. It was found that the Fe-B alloy with Fe_2_B oriented vertically to the corrosion interface possessed the best corrosion resistance to the molten zinc [33,34]. It can be seen that the biggest angle between the interface and the orientation of the borides in the corrosion layer is approximately 90°. The result is consistent with previous studies indicating that the sample with the preferred Fe_2_B growth direction oriented perpendicular to the corrosion interface has the best corrosion resistance.

## 5. Conclusions

The effects of Mn and Al addition on the microstructure and corrosion behaviors of the Fe-3.5B-11W alloys in molten zinc have been investigated in the present work. The results obtained are summarized below:

(1) With the addition of Mn to Fe-3.5B-11W, the microstructures of the Fe-3.5B-11W alloys change from hypoeutectic to eutectic, facilitating the formation of M_2_B-type boride and restraining the formation of M_3_B-type borides. In the meantime, the fracture toughness obviously increases.
(2) Proper Mn and Fe-3.5B-11W contents can stabilize the Fe_2_B phase and improve the corrosion resistance in a zinc bath. The Fe-3.5 wt.% B alloy containing 11 wt.% W, 7wt. % Mn, and 4 wt.% Fe-3.5B-11W, with few microcracks, has the best corrosion resistance.
(3) The lamellar borides provide the mechanical protection for α-(Fe, W, Mn, Al). With the fracture toughness of the borides increasing, the thermal stability of the borides improves, thereby suppressing boride spalling and corrosion failure.

## Figures and Tables

**Table 1:** The nominal chemical composition of Fe-3.5B alloys (wt.%).

Specimens	W	Mn	B	Al	Balance
Fe-3.5B-11W	11	0	3.5	0	Fe
Fe-3.5B-11W-7Mn-1Al	11	7	3.5	1	Fe
Fe-3.5B-11W-7Mn-4Al	11	7	3.5	4	Fe
Fe-3.5B-11W-7Mn-6Al	11	7	3.5	6	Fe
Fe-4.2B-11W-7Mn-4Al	11	7	4.2	4	Fe

**Table 2:** Average composition of the phases in alloys by WDX (at.%) and calculated phase fraction.

Alloy	Phase	Fe	W	B	Mn	Al	Calculated Phase Fraction
Fe-3.5B-11W	α-(Fe, W)	98.7	1.3	-	-	-	64.8%
	(Fe, W)_3_B	75.3	3.0	21.7	-	-	31.4%
	FeWB	34.1	32.3	33.6	-	-	3.8%
Fe-3.5B-11W-7Mn-1Al	α-(Fe, W, Mn, Al)	87.8	0.8	-	5.2	6.2	60.0%
	(Fe, W, Mn)_2_B	52.4	2.6	32.4	12.2	0.4	36.0%
	FeWB	32.2	35.2	32.6	-	-	4.0%
Fe-3.5B-11W-7Mn-4Al	α-(Fe, W, Mn, Al)	82.0	0.7	-	5.5	11.8	28.7%
	(Fe, W, Mn)_2_B	50.7	3.8	32.4	11.6	1.5	71.3%
Fe-3.5B-11W-7Mn-6Al	α-(Fe, W, Mn, Al)	80.2	0.6	-	5.3	13.9	56.6%
	(Fe, W, Mn)_2_B	56.3	3.5	25.7	11.8	2.7	43.4%

**Table 3:** The chemical composition of the phases in the corroded layer of the alloys of different compositions after 48 h of corrosion under the zinc solution at 520 °C by EPMA.

Position	Fe		Al		Zn		W		Mn		Phase
	wt.%	at.%	wt.%	at.%	wt.%	at.%	wt.%	at.%	wt.%	at.%	
+1 in Fe-3.5B-11W	10.9	13.1	-	-	81.4	84.0	7.7	2.5	-	-	δ-FeZn_10_
+2 in Fe-3.5B-11W-7Mn-1Al	11.7	13.7	0.4	1.0	84.1	83.7	3.3	1.2	0.5	0.4	δ-FeZn_10_
+3 in Fe-3.5B-11W-7Mn-4Al	7.1	8.2	-	-	92.9	91.8	-	-	-	-	ζ-FeZn_13_
+4 in Fe-3.5B-11W-7Mn-6Al	31.6	24.1	38.7	61.2	17.7	11.6	11.6	2.7	0.4	0.4	Fe_3_AlZn*_x_*

## Data Availability

The raw/processed data required to reproduce these findings cannot be shared at this time as the data also forms part of an ongoing study.
